# Retinal Vascular and Structural Changes in the Murine Alzheimer’s *APP^NL-F/NL-F^* Model from 6 to 20 Months

**DOI:** 10.3390/biom14070828

**Published:** 2024-07-10

**Authors:** Lidia Sánchez-Puebla, Inés López-Cuenca, Elena Salobrar-García, María González-Jiménez, Alberto Arias-Vázquez, José A. Matamoros, Ana I. Ramírez, José A. Fernández-Albarral, Lorena Elvira-Hurtado, Takaomi C. Saido, Takashi Saito, Carmen Nieto-Vaquero, María I. Cuartero, María A. Moro, Juan J. Salazar, Rosa de Hoz, José M. Ramírez

**Affiliations:** 1 Ramon Castroviejo Institute for Ophthalmic Research, Complutense University of Madrid, 28040 Madrid, Spain; lidsan02@ucm.es (L.S.-P.); inelopez@ucm.es (I.L.-C.); elenasalobrar@med.ucm.es (E.S.-G.); mrgjimenez@ucm.es (M.G.-J.); albearia@ucm.es (A.A.-V.); jomatamo@ucm.es (J.A.M.); airamirez@med.ucm.es (A.I.R.); joseaf08@ucm.es (J.A.F.-A.); marelvir@ucm.es (L.E.-H.); jjsalazar@med.ucm.es (J.J.S.); 2 Health Research Institute of the Hospital Clínico San Carlos (IdISSC), 28040 Madrid, Spain; 3 Department of Immunology, Ophthalmology and ENT, Faculty of Optics and Optometry, Complutense University of Madrid, 28040 Madrid, Spain; 4 Laboratory for Proteolytic Neuroscience, Brain Science Institute, RIKEN, Wako 351-0198, Japan; takaomi.saido@riken.jp; 5 Institute of Brain Science, Faculty of Medical Sciences, Nagoya City University, Nagoya 467-8601, Japan; saito-t@med.nagoya-cu.ac.jp; 6 Centro Nacional de Investigaciones Cardiovasculares (CNIC), Neurovascular Pathophysiology, Cardiovascular Risk Factor and Brain Function Programme, 28029 Madrid, Spain; carmen.nieto@cnic.es (C.N.-V.); mariaangeles.moro@cnic.es (M.A.M.); 7 Hospital 12 de Octubre Research Institute (i + 12), 28029 Madrid, Spain; maricuar@ucm.es; 8 University Institute for Research in Neurochemistry, Complutense University of Madrid (UCM), 28040 Madrid, Spain; 9 Department of Pharmacology and Toxicology, Faculty of Medicine, Complutense University of Madrid (UCM), 28040 Madrid, Spain; 10 Department of Immunology, Ophthalmology and ENT, School of Medicine, Complutense University of Madrid, 28040 Madrid, Spain

**Keywords:** Alzheimer’s disease, *APP^NL-F/NL-F^*, OCTA, OCT, retina

## Abstract

Alzheimer’s disease (AD) may manifest retinal changes preceding brain pathology. A transversal case-control study utilized spectral-domain OCT angiography (SD-OCTA) and Angio-Tool software 0.6a to assess retinal vascular structures and OCT for inner and outer retina thickness in the *APP^NL-F/NL-F^* AD model at 6, 9, 12, 15, 17, and 20 months old. Comparisons to age-matched wild type (WT) were performed. The analysis focused on the three vascular plexuses using AngiooTool and on retinal thickness, which was represented with the Early Treatment Diabetic Retinopathy Study (ETDRS) sectors. Compared to WT, the *APP^NL-F/NL-F^* group exhibited both vascular and structural changes as early as 6 months persisting and evolving at 15, 17, and 20 months. Significant vascular alterations, principally in the superficial vascular complex (SVC), were observed. There was a significant decrease in the vessel area and the total vessel length in SVC, intermediate, and deep capillary plexus. The inner retina in the *APP^NL-F/NL-F^* group predominantly decreased in thickness while the outer retina showed increased thickness in most analyzed time points compared to the control group. There are early vascular and structural retinal changes that precede the cognitive changes, which appear at later stages. Therefore, the natural history of the *APP^NL-F/NL-F^* model may be more similar to human AD than other transgenic models.

## 1. Introduction

Currently, of all murine models employed for the study of Alzheimer’s disease (AD), the *APP^NL-F/NL-F^* model is one of those that most closely reproduces the vascular amyloidosis process that occurs in this disease [1]. This model has normal full-length APP levels while producing significantly higher Aβ_42_ versus Aβ_40_ levels. This overexpression of Aβ_42_ translates into pathological deposits of Aβ in the cerebral cortex and hippocampus. These deposits produce an inflammatory reaction involving microglial and astrocytic infiltration around the deposits starting at 6 months of age [1,2].

It is known that the eye is part of the central nervous system, maintaining functional and structural similarities with it. There is also a relationship between retinal and cerebral vascularization, and both tissues present an immune privilege that allows for the survival of the tissue, as well as the presence of barriers [3,4,5,6].

In pathological conditions and to maintain this immunoprivilege, glial cells must react and therefore become activated [7,8], playing a fundamental role in immunosurveillance and the immune response. One of the fundamental cells for the generation of the blood–brain barrier (BBB) is astrocytes, which induce tight junctions between vascular endothelial cells and thus participate in maintaining the integrity of the BBB and preserving the immune privilege of the CNS [9,10].

The relationship between astrocytes and vessels is already present during angiogenesis, and the development of the mouse retinal vasculature is similar to that of the human retina. The first vessels start from the optic nerve head, preceded by astrocytes [11,12], extending along the inner retinal surface and forming a dense vascular network [13], which is responsible for nourishing the ganglion cells, as well as the upper portion of the inner plexiform layer, constituting the superficial vascular complex (SVC). These vessels are formed through vasculogenesis from angioblasts. From the SVC, branches extend to the lower part of the inner plexiform layer, forming a second vascular network that runs parallel to the first [14,15,16]. This vascular network is formed via angiogenesis, and astrocytes do not precede its formation. This second vascular network nourishes part of the inner nuclear layer as well as the inner portion of the outer plexiform layer, and it is called the deep capillary plexus (DCP). In the mouse, the superficial plexus has been shown to consist mainly of arterioles, which branch into three or four precapillary arterioles. In contrast, the DCP is predominantly venous and consists mainly of capillaries [17].

Third, the intermediate capillary plexus (ICP) develops, which nourishes the inner part of the inner plexiform layer and the superficial portion of the inner nuclear layer. This intermediate vascular plexus is also formed through angiogenesis of the vessels connecting the superficial vascular plexus to the deep vascular plexus [18]. This whole process is completed around the 16th postnatal day, forming an interconnected three-dimensional network that extends throughout the retina [19]. All of these plexuses supply the inner retina, while the outer retina (from the outer limiting membrane to the retinal pigmentary epithelium) is nourished from the choriocapillaris [20,21,22,23,24].

Both in the retina and in the brain, vascular and structural changes that occur with the disease have been described in AD. Vascular changes include the accumulation of Aβ in the blood vessel wall [25], leading to a decrease in blood flow. Over time, this reduced flow may lead to a decrease in vascular density [26], which can differ in severity in the three retinal vascular plexuses as well as in choroidal thickness [27]. These changes in vascular density can be measured in the retina through non-invasive techniques, such as optical coherence tomography angiography (OCTA). This technique allows, without contrast injection, for acquiring an image of the retinal vascular network, which can be analyzed using some image analysis software. This software can provide information on features, such as size, shape, and density of blood flow [28,29,30]. In patients with AD, changes in vascular density and increases in the foveal avascular zone have been observed using this technique, correlating with cognitive decline, and depicting different stages of the disease [31,32,33,34,35].

Concomitant to the vascular changes, thickness changes in the different retinal layers have been described in patients in preclinical stages of the disease, which have been measured through optical coherence tomography (OCT), making these changes an early detection biomarker of AD [36]. Furthermore, in the *APP ^NL-F/NL-F^* model, thickness changes in retinal layers have also been observed, showing thinning and thickening of the total retina thickness from 6 to 20 months of age [37]. However, to the best of our knowledge, this AD model has never been analysed through OCTA.

Therefore, the aim of the present study is to analyze the changes presented in a humanized murine model of AD, such as *APP^NL-F/NL-F^*, in the three retinal vascular layers by conducting a transversal case-control study with OCTA from 6 to 20 months of age, as well as to study the inner and outer retinal thickness through OCT, in order to assess if this AD model could have a natural history similar to the disease evolution in humans.

## 2. Materials and Methods

### 2.1. Animal and Ethics

The study was performed in male *APP^NL-F/NL-F^* mice, which were obtained from the research group led by Dr. Takaomi C. Saito. This mouse carrying the *C57BL/6* mouse genome presents a manipulation in the mouse APP gene using two mutations: *the Swedish mutation* (*NL*), which raises the total amount of Aβ_40_ and Aβ_42_, and the *Beyreuther/Iberian(F)* mutation, which increases the ratio of Aβ_42_/Aβ_40_ [38]. In order for the pathology to develop faster, murine Aβ was deleted, and mice were bred in homozygosis [1]. This means that littermates were not used as control animals, and age-matched *C57BL/6J* mice were used as wild type (WT).

The animals were housed in the animalarium of the School of Medicine of the Complutense University of Madrid, in rooms with controlled lighting conditions (12 h light/dark cycle and light intensity inside of the cages ranging from 9 to 24 lux) and temperature, as well as ad libitum access to food and water.

All procedures were approved by the Animal Welfare Ethics Committee of the Complutense University (PROEX N° 047/16) and reported according to the Association for Research in Vision and Ophthalmology (ARVO) animal use statement. In addition, these procedures were carried out in accordance with European Parliament, Council Directive 2010/63/EU and Spanish legislation (Royal Decree 53/2013).

### 2.2. Experimental Groups

**Figure 1:**
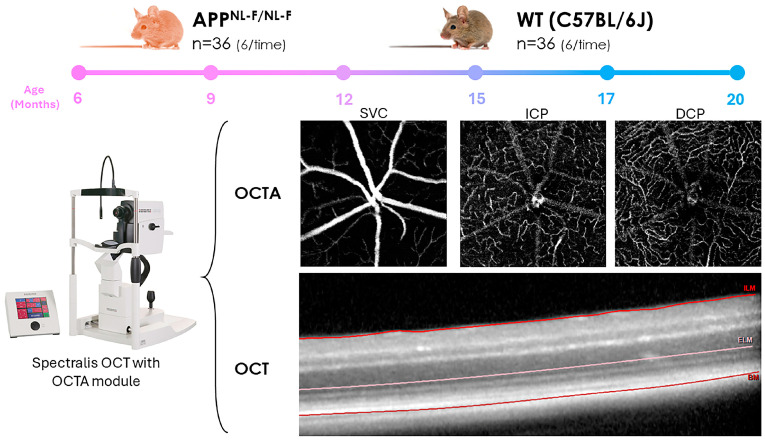
Summary of materials and methods. The upper part shows the study groups and study times. In the lower part are the images of the vascular plexuses obtained by OCTA (SVC: superficial vascular complex, ICP: intermediary capillary plexus and DCP: deep capillary plexus) and the OCT segmentation of the inner and outer retina (ILM: inner limiting membrane, ELM: external limiting membrane, and BM: Bruch’s membrane).

A transversal case-control study was performed in mice at ages 6, 9, 12, 15, 17, and 20 months. Two groups were formed: an experimental group with sample size n = 36, and a control group composed of WT mice, n = 36. At each time point of the study, 6 mice with the *APP^NL-F/NL-F^* genetic variant and 6 WT *C57BL/6J* mice were included (Figure 1).

### 2.3. OCTA and OCT Analysis

OCTA and OCT analysis was performed on the left eye of the mice after anesthesia. An i.p. mixture of medetomidine (0.26 mg/kg; Medetor^®^, Virbac España S.A., Barcelona, Spain) and ketamine (75 mg/kg; Anesketin^®^, Dechra Veterinary Products SLU, Barcelona, Spain) was employed to anesthetize the mice.

After dilation of the mouse pupil using tropicamide (tropicamide 10 mg/ml; tropicamide colircusi, Alcon Healthcare, Barcelona, Spain), the mouse eye was covered with a polymethyl methacrylate contact lens (3.2 mm diameter, base curve 1.7; Cantor&Nissel, Brackley, UK), thus creating a uniform refractive surface. In addition, during the time the animal was anesthetized, it was placed on a thermal blanket to maintain body temperature, and the corneas were hydrated using reticulated artificial tears. All images were centered on the mouse optic nerve, and real-time eye tracking was used in the device’s software to minimize artifacts caused by eye movements or the animal’s breathing.

Retinal vascularization and retinal structure were evaluated using the Spectralis SD-OCT with Heidelberg Eye Explorer v6.13 software (Heidelberg Engineering, Heidelberg, Germany), which has the angiography module. The OCTA acquisition data have been added to Supplementary Table S5.

All OCT images used for the work were checked for off-center artefacts, blurring, shadows, or movements, and therefore no image was discarded. 

The vascular complexes/plexus analyzed were established using Heidelberg software version 1.12.1.0; the superficial vascular complex (SVC), consisting of the vascular plexus of the nerve fiber layer and the superficial vascular plexus, is responsible for nourishing the ganglion cells, as well as the upper portion of the inner plexiform layer. Furthermore, for a deeper understanding, we subdivided the Deep Vascular Complex into the intermediate capillary plexus (ICP) responsible for nourishing the inner part of the inner plexiform layer and the superficial portion of the inner nuclear layer and the deep capillary plexus (DCP) that nourishes part of the inner nuclear layer as well as the inner portion of the outer plexiform layer (Figure 1).

These images were extracted in TIFF format for analysis using AngioTool software (version 0.6a; National Institutes of Health, National Cancer Institute, Bethesda, MD, USA).

The OCT images were also reviewed to ensure that the automatic segmentation of the retinal layers was correct, and those with errors were modified. 

The extraction and representation of the retinal thickness data were performed using ETDRS rings of 1, 2, and 3 mm while discarding the central ring because it was the exit of the large vessels. The 2 and 3 mm rings were divided into four quadrants (superior, inferior, nasal, and temporal). The inner retina was segmented from the inner limiting membrane (ILM) to the external limiting membrane (ELM) and the outer retina from the ELM to Bruch’s membrane. These limits have been described previously by different authors [39,40] (Figure 1). OCT images were required to have a minimum signal-to-noise ratio of 25 dB and an average of 16 B-scans for inclusion. The OCT acquisition data have been added to Supplementary Table S5.

### 2.4. Vascular Analysis with AngioTool

AngioTool (version 0.6a; National Institutes of Health, National Cancer Institute, Bethesda, MD, USA) is a lightweight software that allows for morphometric analysis of several vascular parameters, including vessel area and total number of junctions, branching index, mean vessel length, total number of ends, and lacunarity. This analysis is performed on the full extent of the images taken through OCTA, which were extracted from the Heidelberg image viewer in TIFF format with dimensions of 1660 × 1109 pixels. We cropped the OCTA image, removing the part that belongs to the background image, cropping it to 892 × 889 pixels.

These images show the retinal vasculature in white on a dark background, a feature that makes them compatible with the software. The procedure followed by the analysis has been described by Zudaire et al. [30].

### 2.5. Statistical Analysis

GraphPad Prism 9.0 (GraphPad Software Inc., La Jolla, CA, USA) was used to perform the statistical study. Sample normality was analyzed using the Shapiro–Wilk test. For parametric samples, a Student’s *t*-test was performed for comparison between the case group and the age-matched control group, while the Mann–Whitney U test was used for nonparametric variables. Data were expressed with mean and standard deviation and reported for each study group. Data were considered statistically different at different levels of significance (* *p* < 0.05, ** *p* < 0.01, *** *p* < 0.001, and **** *p* < 0.0001).

### 2.6. Colorimetric Representation

The colorimetric visualization of macular thickness changes between the *APP^NL-F/NL-F^* and WT groups was achieved using the Excel software’s version 2406 build 16.0.17726.20078 color scale function. Regions with no discernible difference are represented in white, areas exhibiting thinning in the *APP^NL-F/NL-F^* related to WT groups are depicted in shades of blue, and those displaying thickening are shown in shades of red. The color intensity is automatically determined by the software in accordance with the magnitude of thickness variation.

## 3. Results

### 3.1. Vascular Analysis of the Superficial Vascular Complex (SVC)

**Figure 2:**
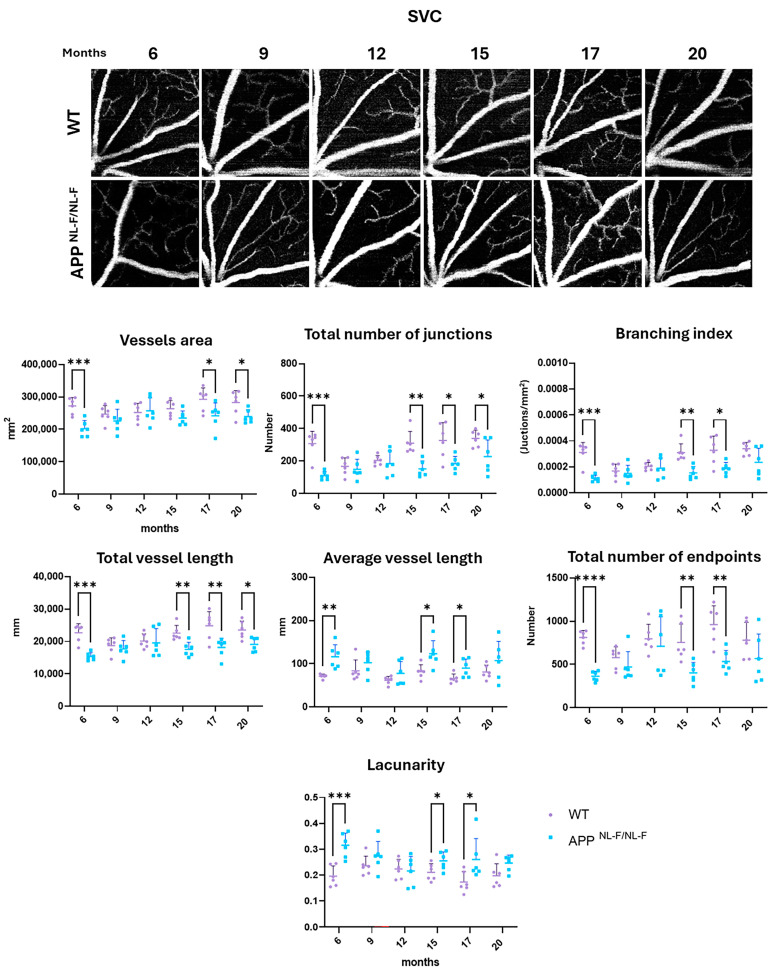
Representative SVC OCTA images and interval scatter plot from WT and *APP^NL-F/NL-F^* mice at 6, 9, 12, 15, 17, and 20 months of age. Vessel area, total number of junctions, branching index, total vessel length, average vessel length, total number of end points, and lacunarity were quantified with AngioTool. * *p* < 0.05, ** *p* < 0.01, *** *p* < 0.001, **** *p* < 0.0001. SVC: superficial vascular complex. (n = 6 for each study group at each time point. The error bars correspond to the standard deviation).

**At 6 months of age**, in the SVC, we observed a significant decrease in all vascular parameters analyzed in the *APP^NL-F/NL-F^* group compared to the WT except for average vessel length (*p*-value 0.029) and lacunarity or vessel non-uniformity (*p*-value 0.0008), which showed a significant increase in transgenic mice compared to controls. We found that the *APP^NL-F/NL-F^* group shows a significant decrease compared to the WT in the following vascular parameters: (i) vessel area (*p*-value 0.0007); (ii) total number of junctions (*p*-value 0.0001); (iii) branching index (*p*-value 0.0001); (iv) total vessel length (*p*-value 0.0001); and (v) total number of end points (*p*-value < 0.0001) (Supplementary Table S1 and Figure 2). 

**At 9 and 12 months of age**, no statistically significant changes were found in the SVC (Supplementary Table S1 and Figure 2). 

**At 15 months of age**, we observed in the SVC a similar behavior of vascular parameters compared to at 6 months, with a significant decrease in most of the vascular parameters analyzed in the *APP^NL-F/NL-F^* group compared to the WT group with the exception of average vessel length (*p*-value 0.0134) and lacunarity or non-uniformity of vessels (*p*-value 0.0471), which presented a significant increase in transgenic mice compared to WT. In this time point, we found that the *APP^NL-F/NL-F^* group in the SVC shows a significant decrease in comparison to the WT group in the following vascular parameters: (i) total number of junctions (*p*-value 0.0012); (ii) branching index (*p*-value 0.0011); (iii) total vessel length (*p*-value 0.0035); and (iv) total number of end points (*p*-value 0.0055) (Supplementary Table S1 and Figure 2). 

**At 17 months of age**, statistically significant changes were observed in the SVC across all of the variables analyzed, consistent with the findings at previous time points. Thus, we found that the *APP^NL-F/NL-F^* group shows a significant decrease with respect to the control group in the following vascular parameters: (i) vessel area (*p*-value 0.0427); (ii) total number of junctions (*p*-value 0.0147); (iii) branching index (*p*-value 0.0157); (v) total vessel length (*p*-value 0.0100); and (vi) total number of end points (*p*-value 0.0020).

However, the average vessel length (*p*-value 0.0287) and lacunarity or vessel non-uniformity (*p*-value 0.0405) presented a significant increase in transgenic mice compared to WT (Supplementary Table S1 and Figure 2). 

**At 20 months of age**, in the SVC, we found that in the *APP^NL-F/NL-F^* group only three vascular parameters were significantly decreased with respect to WT mice: (i) vessel area (*p*-value 0.0325); (ii) total number of junctions (*p*-value 0.0375); and (iii) total vessel length (*p*-value 0.0112) (Supplementary Table S1 and Figure 2).

### 3.2. Vascular Analysis of the Intermediate Capillary Plexus (ICP)

**Figure 3:**
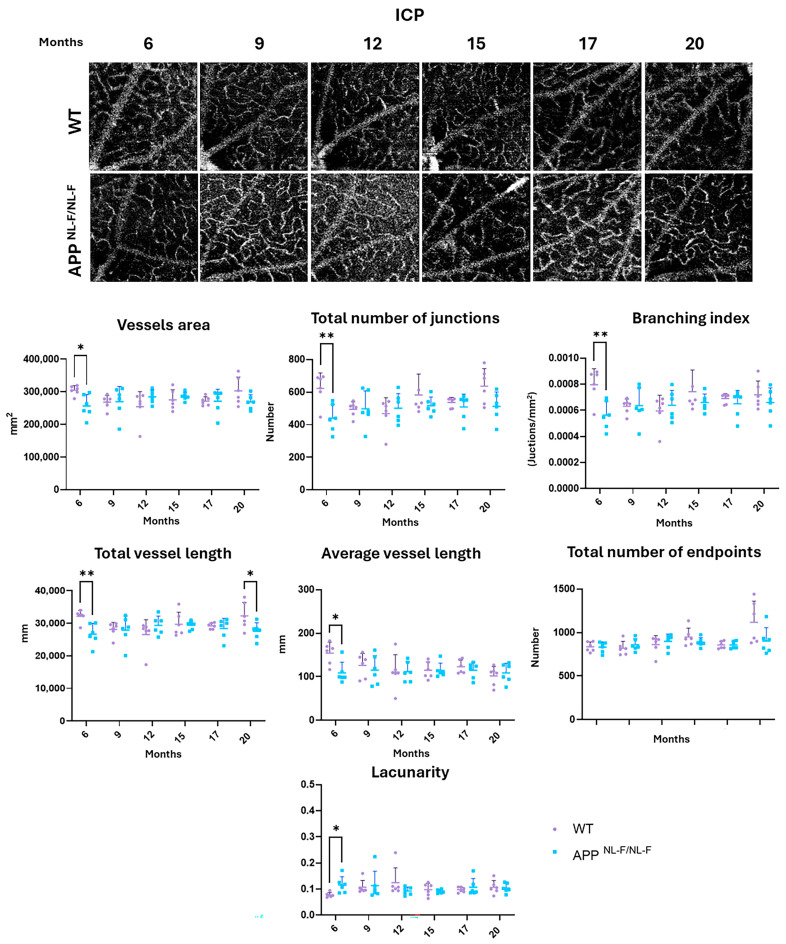
Representative ICP OCTA images and interval scatter plot from WT and *APP^NL-F/NL-F^* mice at 6, 9, 12, 15, 17, and 20 months of age. Vessel area, total number of junctions, branching index, total vessel length, average vessel length, total number of end points, and lacunarity were quantified with AngioTool. * *p* < 0.05, ** *p* < 0.01. ICP: intermediate capillary plexus. (n = 6 for each study group at each time point. The error bars correspond to the standard deviation).

**At 6 months** of age, statistically significant alterations were observed in the ICP across nearly all examined variables, except for the total number of end points in the *APP^NL-F/NL-F^* group compared to the WT group. These alterations manifested as decreases in the following parameters: (i) vessel area (*p*-value 0.0111); (ii) total number of junctions (*p*-value 0.0055); (iii) branching area (*p*-value 0.0057); (iv) total vessel length (*p*-value 0.0050); and (v) average vessel length (*p*-value 0.0102). Additionally, a notable increase in lacunarity was observed (*p*-value 0.0182) (Supplementary Table S2 and Figure 3).

**From 9 to 17 months**, no statistically significant changes were found in any of the vascular parameters analyzed in this plexus, while **at 20 months** only the total vessel length reached statistical significance, being lower in the APP ^NL-F/NL-F^ model than in the WT group (*p*-value 0.0408) (Supplementary Table S2 and Figure 3).

### 3.3. Vascular Analysis of the Deep Capillary Plexus (DCP)

**Figure 4:**
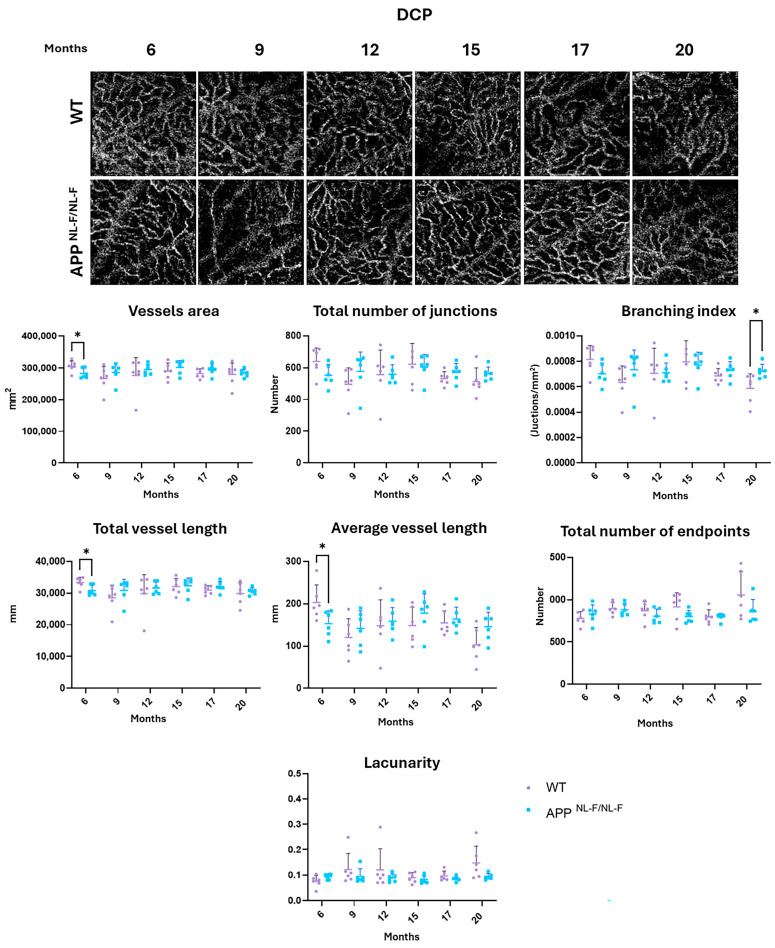
Representative DCP OCTA images and interval scatter plot from WT and APP^NL-F/NL-F^ mice at 6, 9, 12, 15, 17, and 20 months of age. Vessel area, total number of junctions, branching index, total vessel length, average vessel length, total number of end points, and lacunarity were quantified with AngioTool. * *p* < 0.05. DCP: deep capillary plexus. (n = 6 for each study group at each time point. The error bars correspond to the standard deviation).

**At 6 months**, significant differences were observed in certain vascular parameters between the WT group and the *APP^NL-F/NL-F^* group in the DCP. The latter exhibited a decrease in (i) vessel area (*p*-value 0.0431), (ii) total vessel length (*p*-value 0.0274), and (iii) average vessel length (*p*-value 0.0385) (Supplementary Table S3 and Figure 4).

As in the ICP, no vascular parameters were found with statistically significant variations **from 9 to 17 months**, while **at 20 months**, *APP^NL-F/NL-F^* mice showed a statistically higher branching index than WT (*p*-value 0.0264) (Supplementary Table S3 and Figure 4). 

### 3.4. Inner and Outer Retinal Thickness Analysis 

When analyzing the thickness of the outer and inner retina in the APP^NL-F/NLF^ group compared to the WT, we observed a predominant decrease in thickness in the inner retina, while the outer retina showed a predominant increase in thickness at most of the analyzed time points.

**Figure 5:**
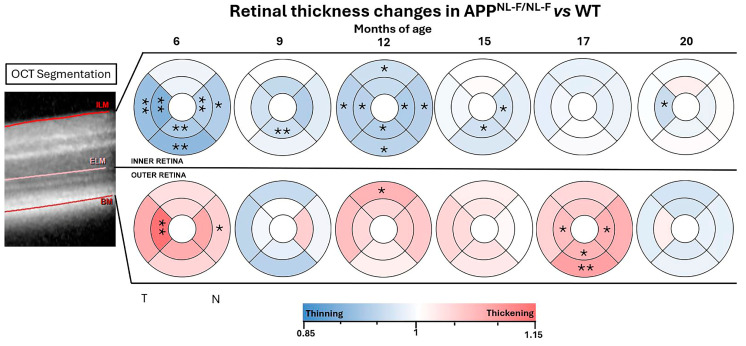
Colorimetric representation of inner and outer retinal thickness differences in each study time point between the *APP^NL-F/NL-F^* and WT groups. ETDRS rings of 1, 2, and 3 mm diameters. Blue tones: thinning. Red tones: thickening. * *p*-value < 0.05, ** *p*-value < 0.01. The central circle has not been analyzed as it corresponds to the exit of the vessels. (ILM: inner limiting membrane, ELM: external limiting membrane, and BM: Bruch’s membrane).

**At 6 months of age**, the *APP^NL-F/NL-F^* group shows a statistically significant inner retinal thickness decrease in the inner and outer rings of the temporal (*p*-values 0.0022 and 0.0043, respectively), inferior (*p*-values 0.0043 in both cases), and nasal sectors (*p*-values 0.0065 and 0.0130) in both cases compared to the WT group (Supplementary Table S4 and Figure 4). However, the outer retina of *APP^NL-F/NL-F^* mice at this time point shows statistically significant thickening in the inner ring in the temporal sector and in the nasal sector of the outer ring related to WT (Supplementary Table S4 and Figure 5).

**At 9 months**, we only found statistically significant differences in the inner retina, with the *APP^NL-F/NL-F^* group showing a significant thinning of the inferior sector of the inner ring in comparison to the WT group (*p*-value 0.0087) (Supplementary Table S4 and Figure 5).

**At 12 months**, the inner retina of the *APP^NL-F/NL-F^* group shows statistically significant thinning in the inner and outer rings in the temporal (*p*-values 0.0173 and 0.0238), inferior (*p*-values 0.0108 and 0.0130), and nasal sectors (*p*-values 0.0108 and 0.0238) and superior sectors of the outer ring (*p*-value 0.0216) when compared to the WT group. However, in the outer retina, the transgenic group shows only a statistically significant increase in thickness in the superior sector of the outer ring compared to the WT group (*p*-value 0.0476) (Supplementary Table S4 and Figure 5).

**At 15 months**, *APP^NL-F/NL-F^* animals show a statistically significant decrease of the inner retina in the inferior and nasal sectors of the inner ring in comparison to the WT group (*p*-values 0.0433 and 0.0260, respectively) (Supplementary Table S4 and Figure 5).

**At 17 months**, there were statistically significant differences only in the outer retina, with the transgenic group showing significant increases in thickness in the nasal (*p*-value 0.0130), temporal (*p*-value 0.0368), and inferior sectors of the inner and the outer ring with respect to the control group (*p*-values 0.0390 and 0.0065, respectively) (Supplementary Table S4 and Figure 5).

Finally, at **20 months**, the transgenic animals show a statistically significant decrease in thickness of the temporal sector of the inner ring in the inner retina compared to the control group (*p*-value 0.0303) (Supplementary Table S4 and Figure 5).

## 4. Discussion

The present transversal case-control study analyzes the retinal vascular and structural changes observed with OCTA and OCT in the *APP^NL-F/NL-F^* transgenic model from 6 to 20 months of age compared with a WT model in the same period of study.

First, it is noteworthy that both vascular and structural changes appear in the retina at the earliest time point of study, 6 months of age. Previous studies have shown that cerebral hypoperfusion generates a neurovascular uncoupling that causes a breakdown of the BBB that occurs early in the development of AD in both patients and animal models [41,42,43,44,45]. Also, numerous vascular abnormalities have been identified in the retina of AD patients, including reduced blood flow and vascular attenuation, changes that together with venular narrowing, loss of pericytes, and accumulation of Aβ in the retinal microvasculature could explain in part the neurodegenerative process [46,47,48,49]. Moreover, postmortem vascular analysis of AD patients has identified that loss of pericytes and vascular platelet-derived growth factor receptor β (PDGFRβ) that accompany increased vascular amyloidosis in the retina, compromise the integrity of the blood retinal barrier, and provide new targets for the diagnosis and therapeutics of AD [50].

Vascular abnormalities have been demonstrated in experimental models of AD; however, most studies have been carried out using techniques other than OCTA to demonstrate these changes. These histological techniques require tissue processing, as the incidence of errors in the processing quite significant [51] and therefore errors may occur in the analytical and postanalytical phases [52]. In the *APP/PS1* model, a capillary degeneration between 4 and 8 months of age was observed ex vivo through the isolation and staining of retinal vessels, presenting a strong association with the lack of PDGFRβ simultaneous with the presence of Aβ deposits in these capillaries [53]. Also early, at 3 months of age in a murine model of acrolein-induced AD, retinal venous beading has been observed through fundus fluorescence angiography [54]. In *5xFAD* mice, this technique has revealed an increase in venous caliber alongside a decrease in venular flow velocity. Moreover, analysis using fluorescent microspheres has indicated an increase in oxygen levels in both veins and arteries [55]. Similarly, in the *APP^NL-G-F^* transgenic model, characterized by three mutations, a decrease in venule diameter, not seen in arterioles, coincides with an increased Aβ load at 18 months of age, as observed through retinal whole-mounts stained with isolectin B4 [56].

Many of these changes are concordant with the structural alterations found in the cerebral microvasculature, such as deformation and loss of vessels accompanied by small vascular deposits, at early ages (approximately 3 months) of the *APP23tg* model [57].

The *APP^NL-F/NL-F^* murine model used in this work compared to first-generation Alzheimer’s models has several advantages, presenting a less altered physiology and a less artificial phenotype. This occurs because it maintains normal levels of full-length APP, and the resultant products from its cleavage generate significantly more Aβ_42_ compared to other mouse models overexpressing APP or control mice. It also has a significantly higher Aβ_42_/Aβ_40_ ratio [38]. This higher proportion of Aβ_42_ in the *APP^NL-F/NL-F^* model causes the development of pathological Aβ deposits in the cerebral cortex and hippocampus, which in turn triggers the infiltration of microglia and astrocytes surrounding Aβ plaques from 6 months of age onwards [1]. These changes lead to age-related neurological alterations, such as synaptic disorders and memory impairment [38]. In addition, this model reproduces several pathological features observed in AD patients, suggesting its usefulness as a mouse preclinical model of AD to study the pathological role of amyloidosis in neuroinflammation in this disease [2]. On the other hand, OCTA allows us to analyze changes in retinal vascular parameters in vivo in a non-invasive way, as it is not necessary to inject contrasts, and these can also be analyzed using image analysis software, allowing for research at different stages of the disease [30]. 

In the *APP^NL-F/NL-F^* murine model, at 6 months of age, there is a decrease in the area occupied by vessels in the SVC, ICP, and DCP, as reported in AD patients from preclinical stages [32,49,58]. The SVC present a decrease in total vessel length and an increase in total mean vessel length. The total length decreases because of the loss of branches, which causes the number of vessels to decrease, thus increasing the average vessel length. In addition, at this time point, we observed a decrease in the total number of junctions and a decrease in the branching index in the SVC and ICP plexuses. There is also a decrease in the number of end points and an increase in lacunarity in the SVC. It should be noted that in the *APP^NL-F/NL-F^* model, most of the changes are observed in the SVC, as is the case in patients with mild cognitive impairment [59], and, contrary to other murine models of tauopathy, where most of the changes occur in ICP and DCP [60]. This SVC comprises larger vessels that supply blood to the ganglion cell layer and the innermost part of the IPL, both integral components of the inner retina. At this specific time point in our study involving *APP^NL-F/NL-F^*, a statistically significant decrease in thickness was observed in these layers. A possible explanation could be the loss of retinal neurons caused by changes in vessel dynamics, which has been demonstrated by studies of neurovascular coupling in patients with mild cognitive impairment [61].

One of the vascular parameters analyzed in this study is lacunarity, which measures the distribution of space or pixel dispersion within a retinal image [62,63]. Therefore, this parameter characterizes the heterogeneity of the pixels within an image, as the greater the heterogeneity of the vascular plexus the higher the lacunarity. Our results show that in SVC, the lacunarity is higher in the transgenic model analyzed than in the WT, reaching significance at 6, 15, and 17 months. We also found that in the ICP, the lacunarity is higher in the *APP^NL-F/NL-F^* model than in the WT at 6 months. This indicates that changes in the vascular network are already occurring at very early stages, which would be compatible with functional alterations of the vasculature, which occur as the neurodegenerative process progresses. Moreover, in studies of patients with MCI, retinal vascular parameters showed a smaller fractal dimension and a larger lacunarity [64,65,66,67,68]. It is striking to observe how the initial changes emerge at 6 months and do not become significant again until 15 months. The fact that the perfusion of the vessels in the SVC is only decreased in *APP^NL^-^F/NL-F^* mice at 6 months of age, although it appears similar to that of WT mice at 9 and 12 months of age, could be attributed to two situations. On the one hand, the increase in the area occupied by the vessels could be due to an inflammatory process with hypoxia that causes an increase in retinal blood flow and microvessels that are normally not detected with OCTA due to low blood flow below the detection level, which would become visible [69]. On the other hand, it could coincide with the opening of arteriovenous shunts that would act as a compensatory mechanism to the acute ischaemia observed in the first months of the study [58,70]. In other AD models, such as the *5xFAD* model, it has also demonstrated by OCTA significant vascular narrowing and a trend of decreased capillary density at 6 months of age [71], at which time this model already shows behavioral alterations [72] and neuronal death [73]. In the *3xTg-AD* model, very early changes in vascular flow in arterioles and venules have also been reported before 5 months of age [57], concomitant with cognitive changes that appear between 3 and 5 months of age [74]. However, the model analyzed in the present work develops memory dysfunction at 18 months of age [1,75], thus being able to observe vascular changes early in stages where cognitive symptomatology has not yet appeared.

In the murine model studied in this work, Aβ deposits and dystrophic neurites begin to develop at 6 months of age in the cortex and hippocampus [76], brain areas that are affected early in AD patients [77], while neurofibrillary tangles (NFTs) and neurophil threads (NTs) do not form [38]. In the brain, it has been shown that Aβ_42_ fragments (insoluble) are deposited in extracellular amyloid plaques, which exert adverse vascular effects by increasing oxidative stress, inflammation, and cell apoptosis [45,78]. However, Aβ_40_ fragments (soluble) aggregate in the perivascular space around arterioles and in the wall of arteries entering arterioles, capillaries, and, occasionally, veins [45,79]. Also, Aβ is known to reduce capillary blood flow by increasing oxidative stress and causing the release of endothelin-1 (ET1), which constricts capillary pericytes [44,80]. Another form in which Aβ can be found are oligomers, which are accumulations of soluble Aβ peptides and considered the most toxic form of Aβ [81]. In the human retina, in a possible preclinical stage of the disease, it is known that Aβ oligomers accumulate to a threshold above which the disease begins to be symptomatic [82]. These oligomers begin neuropathological processes, such as neuroinflammation, leading to an increasing number of neurons with limited functionality, which progressively die, ultimately causing neurodegeneration [82].

All of these pathophysiological mechanisms that produce Aβ deposits could explain that in our study with the *APP^NL-F/NL-F^* model we found at 6 months a decrease in all vascular parameters, except for mean total vessel length and lacunarity. Furthermore, in this model of AD, we observed a statistically significant thickness decrease in the inner retina, where Aβ_42_ is predominantly localized in AD [83]. 

Considering that 6 months in the mouse would be equivalent to 20 years in the human [84], we are aware of the precocity of the alterations, but we cannot forget that we are performing a study in a mouse model containing familial AD mutations [38,85].

In the KI *APP^NL-F/NL-F^* mouse model in the SVC, we also found vascular alterations at 15, 17, and 20 months of age. In these months, as well as at 12 months, there are also thickness decreases in the inner retina, while in the outer retina there are more areas with thickness increases that reach statistical significance. In the *APP^NL-F/NL-F^* murine model of AD, it was observed that at 3 months of age 50% of retinal microglia contained a ligand specific for PHF-1 (P-tau variant) and for AT8 + aggregates increasing significantly in these mice at 9 and 12 months of age [59]. This process could explain the increased thickness found in our work in the outer retina at times when the inner retina shows a greater neurodegenerative process. This fact could be supported by a recent proteomics study in retinas of AD patients demonstrating the activation of specific inflammatory and neurodegenerative processes [83], which could explain the changes in retinal thickness observed in this work.

To the best of our knowledge, this is the first work that analyzes in vivo retinal vascularization in the *APP^NL-F/NL-F^* murine model using OCTA. Among the news of this work are the existence of retinal vascular and structural changes at 6 months of age, an early stage in the evolution of the APP model. In this stage, Aβ deposits and dystrophic neurites begin to develop in the cortex and hippocampus [76], but cognitive disorders have not yet appeared. All of this makes this model similar in natural history to the human evolution of AD compared to other transgenic models. Our study, together with numerous clinical papers, demonstrates that new technologies, such as OCT and OCTA, have a great capacity for early detection of retinal vascular and tissue changes in AD and could help in monitoring the disease [86,87,88,89]. OCTA allows for acquiring images of the retinal vasculature like those of conventional angiography, but with many advantages, as it allows for acquiring images without using contrasts and obtaining cross-sectional images of the retinal morphology and the architecture of the vessels. However, our work is not without weaknesses. First, OCTA images do not allow for the analysis of the peripheral retinal vascularization, thus losing part of the information [90]. In addition, the images sometimes present artifacts that make them unanalyzable using image processing programs, especially of the deepest vascular plexuses, so that future improvements in image acquisition quality and OCTA data processing would be necessary to overcome these limitations [91]. Finally, these data should be corroborated with longitudinal studies, where the same animals would be longitudinally analyzed from 6 to 20 months to know the changes evolution in vascular parameters over time.

## 5. Conclusions

In conclusion, the *APP^NL-F/NL-F^* murine model shows early alterations in the retinal vascular network at 6 months of age that are maintained and evolve at 15, 17, and 20 months and are mainly concentrated in the SVC. These changes are concomitant with a thinning of the inner retinal layers at 6, 12, and 15 months of age that may be due to neurodegenerative processes. On the other hand, thickening of the outer retinal layers, mainly at 6 and 17 months of age, could represent a neuroinflammatory process. There are early retinal vascular and structural changes that precede the cognitive changes, which appear at later stages, and the evolution of the *APP^NL-F/NL-F^* model may be more similar to human AD than other transgenic models.

## Data Availability

Data are available upon request from the corresponding author.

## Supplementary Materials

The following supporting information can be downloaded at: https://www.mdpi.com/article/10.3390/biom14070828/s1, Table S1: Date analysis of SVC at the different study times. Table S2: Date analysis of ICP at the different study times. Table S3: Analysis date of DVP at the different study times. Table S4: Analysis date of retinal thickness at the different study times. Table S5: OCTA and OCT adquisition parameters.
