# Supplementary material for: Retinal Vascular and Structural Changes in the Murine Alzheimer’s APPNL-F/NL-F Model from 6 to 20 Months
Source: Biomolecules. 2024 Jul 10;14(7):828. doi: 10.3390/biom14070828 (PMC11274728; doi:10.3390/biom14070828)
Supplement: Supplementary file 1 [file biomolecules-14-00828-s001.zip › Supplementary Table 1.pdf]

Supplementary Table 1. Date analysis of SVC at the different study times.(WT: wild type, n= 6 for each study group at each time point).

		WT n=6					APP$^{NL-F/NL-F}$ n=6					
		**Mean**	**SD**	**IR**	**Min**	**Max**	**Mean**	**SD**	**IR**	**Min**	**Max**	**P-value**
**6 Months**	**Vessels area**	272326	25383	(244116-295830)	237619	297736	202128	24585	(177805-223489)	177644.000	241871.00	**0.0007*** **
	**Total number of junctions**	307.70	75.75	(264.8-353.3)	159	360	110.00	25.01	(86.25-132.8)	84.000	150.00	**0.0001*** **
	**Branching index**	0.0003108	0.00007747	(0.000269-0.000361)	0.000158	0.000361	0.0001108	0.00002475	(0.00008696-0.0001333)	0.00008526	0.0001502	**0.0001*** **
	**Total vessels length**	22690	2756	(19911-24470)	18044	24705	15388	1202	(14333-16408)	14052.000	17359.00	**0.0001*** **
	**Average vessels length**	71.14	4.868	(68.99-73.97)	62.01	76.59	116.10	27.72	(92.8-137.8)	88.31	160.7	**0.0290** **
	**Total number of end points**	812.20	79.66	(733.8-881.3)	688	888	362.70	58.00	(311-416.8)	287.000	428.00	**<0.0001**** **
	**Lacunarity**	0.1961	0.03945	(0.1587-0.2431)	0.1547	0.2448	0.3151	0.0473	(0.2657-0.3625)	0.2534	0.3697	**0.0008*** **

**9 Months**	**Vessels area**	246989	25857	(230488-267213)	203279	278244	225950	36093	(196445-248245)	178980.000	285789.00	0.2727
	**Total number of junctions**	166.70	53.27	(119-220.50)	86	222	149.20	61.93	(113-199.5)	74.000	255.00	0.6112
	**Branching index**	0.0001677	0.00005316	(0.0001209-0.000223)	0.0000874	0.000223	0.0001499	0.00006221	(0.0001138-0.0001986)	0.00007401	0.0002571	0.6047
	**Total vessels length**	18749	2410	(16715-20627)	14544	21122	17730	2576	(16056-19323)	13802.000	21659.00	0.4954
	**Average vessels length**	83.51	25.33	(67.87-93.6)	65.07	133.9	102.70	24.44	(80.53-122.4)	61.71	127.3	0.2113
	**Total number of end points**	579	122.3	(439.0-669.8)	406	714	470	178.2	(367.3-544.5)	356.000	825.00	0.2449
	**Lacunarity**	0.2359	0.03752	(0.2051-0.2569)	0.199	0.3051	0.2734	0.05712	(0.235-0.3037)	0.1944	0.3703	0.2083
**12 Months**	**Vessels area**	252317	27804	(226808-280010)	214650	282687	257094	40355	(227563-302661)	204510.000	309768.00	0.8161
	**Total number of junctions**	203.30	30.22	(176.5-230)	169	251	184.50	77.94	(106.8-268.8)	97.000	289.00	0.5932

	**Branching index**	0.0002037	0.0000297	(0.000176-0.000248)	0.00017	0.000248	0.0001894	0.00007844	(0.0001115-0.0002747)	0.00009926	0.0002929	0.6849
	**Total vessels length**	20109	2226	(18268-22329)	17502	23478	19591	4356	(15703-24849)	15656.000	25304.00	0.8004
	**Average vessels length**	61.72	9.16	(53.99-68.17)	46.01	72.03	77.77	27.70	(54.34-109.7)	53.05	112.6	0.2074
	**Total number of end points**	796.80	167.80	(678.5-924.3)	596	1084	711.00	339.80	(416.8-1078)	374.000	1118.00	0.5912
	**Lacunarity**	0.2237	0.03703	(0.1843-0.26)	0.1815	0.2734	0.2161	0.05564	(0.2734-0.2161)	0.1476	0.2828	0.786127
**15 Months**	**Vessels area**	263480	25376	(239572-286547)	232381	295362	234731	22100	(215138-271906)	213680.000	271906.00	0.0628
	**Total number of junctions**	310.30	71.67	(263.8-346.5)	260	450	152.20	48.84	(113-201.3)	104.000	229.00	**0.0012****
	**Branching index**	0.0003097	0.00006846	(0.0002665-0.000443)	0.000259	0.000443	0.0001538	0.0000486	(0.0001142-0.0002028)	0.000105	0.0002297	**0.0011****
	**Total vessels length**	22570	2381	(21061-24247)	20651	27079	17544	2198	(15775-19187)	15069.000	21111.00	**0.0035****

	**Average vessels length**	81.62	16.21	(69.57-90.04)	59.29	108.7	123.30	29.97	(104.4-142.2)	84.32	175.2	**0.0134***
	**Total number of end points**	754.70	212.40	(597.3-998.3)	529	1059	402.70	120.90	(300.3-513.3)	244.000	571.00	**0.0055****
	**Lacunarity**	0.2107	0.03275	(0.1837-0.2409)	0.1722	0.2562	0.2549	0.03489	(0.2234-0.2895)	0.2066	0.2942	**0.0471***
**17 Months**	**Vessels area**	292388	35515	(253020-316285)	241830	335186	241906	39704	(212041-266572)	172004.000	288456.00	**0.0427***
	**Total number of junctions**	326.70	110.00	(220.8-434.3)	163	441	183.50	45.75	(145.5-220)	123.000	256.00	**0.0147***
	**Branching index**	0.000328	0.0001095	(0.0002228-0.000444)	0.000165	0.000444	0.000187	0.00004652	(0.0001481-0.0002211)	0.0001268	0.000263	**0.0157***
	**Total vessels length**	24850	4341	(20482-28071)	18287	30157	18139	2830	(15682-19919)	13097.000	20648.00	**0.0100****
	**Average vessels length**	65.78	10.44	(58.29-71.85)	54.39	84.96	89.95	20.69	(68.79-112.2)	68.37	114.1	**0.0287***
	**Total number of end points**	960.20	217.40	(755-1133)	644	1222	534.80	125.80	(441-654)	390.000	735.00	**0.0020****

	**Lacunarity**	0.1731	0.04033	(0.1447-0.217)	0.1249	0.2314	0.2601	0.0812	(0.2118-0.3151)	0.2019	0.416	**0.0405***
**20 Months**	**Vessels area**	282496	37035	(247817-314493)	221531	317181	239541	20686	(222342-260971)	219673.000	272135.00	**0.0325***
	**Total number of junctions**	339.70	49.94	(284.8-382.8)	278	400	227.30	103.30	(134.8-344)	104.000	344.00	**0.0375***
	**Branching index**	0.0003395	0.00004861	(0.0002863-0.000398)	0.000165	0.000398	0.0002342	0.0001092	(0.000398-0.0002342)	0.0001086	0.0003607	0.0564
	**Total vessels length**	23560	2764	(21177-26312)	20054	27356	19113	2168	(16781-21031)	16693.000	21258.00	**0.0112***
	**Average vessels length**	80.83	16.22	(69.05-96.89)	60.24	105.1	107.40	44.37	(64.21-142.7)	49.79	173.3	0.1980
	**Total number of end points**	780.50	206.60	(560.8-1009)	557	1039	567.80	284.10	(315.8-839.5)	303.000	1021.00	0.1689
	**Lacunarity**	0.1973	0.04669	(0.1587-0.2282)	0.1556	0.2796	0.2464	0.03077	(0.2153-0.2693)	0.1963	0.2741	0.0571
