# Supplementary material for: Retinal Vascular and Structural Changes in the Murine Alzheimer’s APPNL-F/NL-F Model from 6 to 20 Months
Source: Biomolecules. 2024 Jul 10;14(7):828. doi: 10.3390/biom14070828 (PMC11274728; doi:10.3390/biom14070828)
Supplement: Supplementary file 1 [file biomolecules-14-00828-s001.zip › Supplementary table 2.pdf]

Supplementary table 2. Date analysis of ICP at the different study times.(WT: wild type, n= 6 for each study group at each time point).

		WT n=6					$APP^{NL\text{-}F/NL\text{-}F}$ n=6					
		**Mean**	**SD**	**IR**	**Min**	**Max**	**Mean**	**SD**	**IR**	**Min**	**Max**	**P-value**
**6 Months**	**Vessels area**	304669	14572	(294300-316722)	279145	319825	256666	34889	(231042-292989)	205296	297749	**0.0111***
	**Total number of junctions**	623.00	94.00	(563,3-690,3)	447	694	440.50	82.25	(362-530,8)	326	548	**0.0055****
	**Branching index**	0.0007961	0.0001224	(0,0007155-0,0008806)	0,0005683	0,0008947	0.0005627	0.0005627	(0,0004626-0,0006799)	0,00042	0,0006947	**0.0057****
	**Total vessels length**	32139	1832	(24361-29990)	21248	30090	26624	3293	(31375-33154)	28605	33987	**0.0050****
	**Average vessels length**	154.10	25.12	(127,5-172,9)	116,3	182,6	108.80	24.61	(95,93-122,4)	87,8	156,7	**0.0102***
	**Total number of end points**	835.00	55.59	(789,3-896)	766	899	831.30	60.78	(768,8-881,5)	738	889	0.9153
	**Lacunarity**	0.07766	0.009156	(0,07061-0,08336)	0,06815	0,09438	0.1156	0.03166	(0,08622-0,1368)	0,08485	0,1709	**0.0182***

**9 Months**	**Vessels area**	268352	20699	(252718-282032)	232710	292737	270284	46671	(233856-307098)	185977	310119	0.9280
	**Total number of junctions**	495.20	45.51	(454,3-525,3)	419	547	497.70	109.80	(430-616)	328	625	0.9599
	**Branching index**	0.0006309	0.00005631	(0,0005811-0,0006674)	0,0005352	0,0006938	0.0006359	0.0001389	(0,0005539-0,0007814)	0,0004187	0,0008012	0.9365
	**Total vessels length**	28071	2186	(24902-31420)	20039	32390	27877	4363	(26677-29627)	23932	29874	0.9243
	**Average vessels length**	126.00	27.30	(93,59-147,7)	90,31	156,3	115.00	33.44	(81,46-149,3)	77,97	161,1	0.5484
	**Total number of end points**	819.50	78.74	(748,5-871,5)	744	960	858.80	69.57	(809,5-928,8)	772	964	0.3807
	**Lacunarity**	0.107	0.02623	(0,09332-0,1176)	0,09084	0,1598	0.1131	0.05566	(0,08103-0,1394)	0,07704	0,2239	0.8146
**12 Months**	**Vessels area**	254429	46534	(231388-286710)	163379	288831	284943	20434	(266878-303108)	255897	311768	0.1720
	**Total number of junctions**	468.20	97.30	(412,8-533,3)	280	543	501.20	90.05	(419,5-579,5)	397	629	0.5556

	**Branching index**	0.0005952	0.000121	(0,000526-0,0006753)	0,000361	0,000688	0.0006388	0.0001143	(0,0005353-0,000741)	0,000506	0,000798	0.5349
	**Total vessels length**	26524	4566	(26909-31464)	25751	33523	29332	2850	(25061-28954)	17293	29285	0.2301
	**Average vessels length**	111.00	39.73	(92,88-130,9)	49,84	175	111.70	22.46	(88,26-135,4)	88,05	142,5	0.9699
	**Total number of end points**	862.00	102.50	(790-928,8)	667	931	897.50	83.89	(813,3-961)	760	973	0.5263
	**Lacunarity**	0.1242	0.05692	(0,09343-0,1426)	0,09337	0,2395	0.09239	0.01505	(0,07858-0,1063)	0,0733	0,1119	0.2146
**15 Months**	**Vessels area**	275628	30749	(249669-306280)	239927	321953	286265	12442	(277555-296152)	267978	305165	0.4504
	**Total number of junctions**	582.30	129.60	(501,3-667,5)	543	831	516.80	53.34	(475-556,5)	448	603	0.2789
	**Branching index**	0.0007432	0.0001675	(0,0006393-0,000854)	0,00061	0,001064	0.0006593	0.00006695	(0,000606-0,00071)	0,000573	0,000767	0.2815
	**Total vessels length**	29694	3698	(27940-30306)	27556	30943	29371	1291	(26864-33054)	26306	35881	0.844

	**Average vessels length**	114.60	19.20	(99,60-134,90)	91,98	140,8	114.30	16.79	(103,7-122)	100,8	147,3	0.9801
	**Total number of end points**	947.80	103.20	(863,3-1018)	849	1136	887.50	52.29	(855,3-927,5)	817	974	0.2303
	**Lacunarity**	0.09773	0.0242	(0,07439-0,1179)	0,06408	0,1261	0.09035	0.006998	(0,08455-0,09797)	0,08295	0,1007	0.4895
	**Vessels area**	271577	13125	(258902-282307)	257289	292001	271048	36852	(239697-296297)	204639	296965	0.9742
	**Total number of junctions**	537.00	30.36	(498,5-559)	497	559	509.20	79.03	(431,3-559,8)	375	574	0.4394
**17 Months**	**Branching index**	0.0006902	0.0000379 9	(0,0006428-0,0007159)	0,0006385	0,0007206	0.0006508	0.0001022	(0,0005491-0,0007178)	0,0004784	0,0007355	0.3974
	**Total vessels length**	29190	902.30	(27940-30306)	23102	31156	28400	3088	(28153-30071)	28135	30253	0.5608
	**Average vessels length**	122.80	15.53	(109.2-140,3)	108,2	142,9	114.50	18.48	(94,73-128,4)	86,2	133,3	0.4191

	**Total number of end points**	893.80	78.84	(828,0-942.50)	741	950	856.50	41.00	(822,8-901,5)	813	909	0.4680
	**Lacunarity**	0.09733	0.009878	(0,08808-0,1076)	0,08395	0,1076	0.1069	0.03317	(0,08569-0,1312)	0,08546	0,1698	0.5132
**20 Months**	**Vessels area**	303020	42041	(267042-346894)	254887	363347	268725	22203	(247268-287472)	241163	302469	0.1077
	**Branching index**	636.20	108.20	(522,3-729)	497	559	512.50	87.78	(440,8-589)	371	619	0.0548
	**Junctions density**	0.0007193	0.0001062	(0,000637-0,0008103)	0,00061	0,000901	0.0006595	0.0001117	(0,0005663-0,000756)	0,00048	0,000795	0.3642
	**Total vessels length**	32263	4164	(25372-29106)	23735	31259	27586	2544	(28930-36667)	26941	38018	**0.0408***
	**Average vessels length**	101.60	21.68	(78.46-116,1)	69,13	127	108.60	22.20	(89,62-128,2)	75,9	131,9	0.5892
	**Total number of end points**	1118	243.50	(894,8-1360)	882	1442	901.80	153.60	(1442-901.80)	762	1183	0.0962
	**Lacunarity**	0.1064	0.026	(0,0884-0,1246)	0,07349	0,151	0.1038	0.01806	(0,09106-0,1235)	0,07802	0,1263	0.8415
