# Supplementary material for: Retinal Vascular and Structural Changes in the Murine Alzheimer’s APPNL-F/NL-F Model from 6 to 20 Months
Source: Biomolecules. 2024 Jul 10;14(7):828. doi: 10.3390/biom14070828 (PMC11274728; doi:10.3390/biom14070828)
Supplement: Supplementary file 1 [file biomolecules-14-00828-s001.zip › Supplementary Table 3.pdf]

Supplementary Table 3. Analysis date of DVP at the different study times.(WT: wild type, n= 6 for each study group at each time point).

		WT n=6					APP$^{NL-F/NL-F}$ n=6					
		**Mean**	**SD**	**IR**	**Min**	**Max**	**Mean**	**SD**	**IR**	**Min**	**Max**	**P-value**
**6 Months**	**Vessels area**	304793	17255	(294454-317094)	275561	328026	282644	15847	(269578-302117)	268647	303234	**0.0431***
	**Total number of junctions**	639.30	84.28	(576,5-710,3)	497	717	552.00	68.86	(503,5-619,8)	454	646	0.0777
	**Branching index**	0.0008156	0.0001085	(0,0007334-0,0009062)	0,0006338	0,0009129	0.0007026	0.00008598	(0,0006401-0,0007875)	0,0005784	0,0008179	0.0734
	**Total vessels length**	33318	1675	(32133-34633)	30315	34906	30835	1660	(29478-32933)	29361	32950	**0.0274***
	**Average vessels length**	203.20	41.63	(172,1-232,8)	160,4	278,6	153.20	30.11	(124,4-180,8)	110,5	183,3	**0.0385***
	**Total number of end points**	778.70	80.96	(722-857,3)	653	879	830.30	107.80	(747,5-901,5)	662	984	0.3700

	**Lacunarity**	0.07491	0.02272	(0,05983-0,09088)	0,03567	0,1037	0.09312	0.01061	(0,08038-0,1022)	0,07941	0,1023	0.105603
**9 Months**	**Vessels area**	266028	38375	(239049-295609)	199048	310902	285631	29328	(268111-304382)	230217	313086	0.3436
	**Total number of junctions**	496.20	103.00	(422,8-585,8)	311	591	577.00	121.90	(491,3-653,5)	345	661	0.2431
	**Branching index**	0.0006337	0.0001317	(0,0005407-0,0007467)	0,0003962	0,0007558	0.0007352	0.000154	(0,0006298-0,000834)	0,0004406	0,0008368	0.2478
	**Total vessels length**	28479	4032	(25952-31571)	20951	32212	30884	3496	(28213-33201)	24333	33262	0.2956
	**Average vessels length**	120.30	44.31	(84,03-159,3)	64,46	187,3	142.10	41.33	(98,22-178,5)	86,6	190,1	0.3985
	**Total number of end points**	897.30	75.36	(835,8-976,5)	796	999	882.00	71.39	(820,3-957)	812	990	0.7250
	**Lacunarity**	0.1214	0.06389	(0,08193-0,1475)	0,07767	0,2487	0.09469	0.02961	(0,07677-0,1057)	0,07593	0,1539	0.3746
	**Vessels area**	276049	55813	(252365-316488)	166548	316654	294813	16716	(279766-310980)	276836	319217	0.4485

**12 Months**	**Total number of junctions**	556.00	155.80	(459,5-644,8)	275	743	558.80	60.61	(506-604)	506	667	0.9677
	**Branching index**	0.0007063	0.0001965	(0,0005829-0,0008164)	0,0003536	0,0009448	0.0007107	0.00007706	(0,0006446-0,0007675)	0,0006434	0,0008489	0.9601
	**Total vessels length**	29838	6005	(26984-34168)	18107	34344	31608	1941	(29778-33883)	29681	33902	0.5077
	**Average vessels length**	148.00	61.52	(108,7-185,2)	47,52	236,9	158.50	32.44	(134,6-186,1)	114,6	209,3	0.7192
	**Total number of end points**	869.50	108.30	(786-956,5)	681	988	805.20	84.02	(728,8-902,5)	725	916	0.2771
	**Lacunarity**	0.1199	0.08396	(0,07038-0,1485)	0,0698	0,2889	0.0886	0.01606	(0,07373-0,1011)	0,07063	0,1138	0.3913
**15 Months**	**Vessels area**	289744	25496	(266680-311904)	254153	326107	301703	21567	(279830-319716)	267564	321312	0.4010
	**Total number of junctions**	622.20	129.70	(487,5-728,3)	459	804	604.50	78.72	(556,3-669,5)	458	674	0.7813

	**Branching index**	0.0007967	0.000166	(0,0006228-0,0009298)	0,000586	0,001028	0.0007718	0.0001005	(0,0007093-0,000852)	0,000584	0,000861	0.7604
	**Total vessels length**	32087	2571	(29930-34512)	28616	35644	32316	2453	(30189-34051)	27984	34500	0.8775
	**Average vessels length**	148.50	43.04	(111,5-195,5)	98,34	203,5	178.20	45.13	(143,1-209,8)	98,88	228,5	0.2714
	**Total number of end points**	915.80	165.60	(741,8-1053)	654	1063	800.00	73.10	(739-854,3)	724	915	0.1481
	**Lacunarity**	0.08893	0.01997	(0,07067-0,1092)	0,06127	0,1122	0.08236	0.01623	(0,06935-0,09956)	0,06715	0,1075	0.5461
**17 Months**	**Vessels area**	282214	14538	(270609-297339)	262314	298621	294765	17698	(282634-307795)	265529	318837	0.2092
	**Total number of junctions**	531.30	43.71	(498,5-561,3)	473	601	570.70	55.68	(524,5-612)	484	648	0.2034
	**Branching index**	0.0006851	0.0000586 6	(0,0006429-0,0007269)	0,000618	0,0007844	0.0007293	0.0000689 8	(0,0006725-0,0007798)	0,000623	0,000827	0.2587

	**Total vessels length**	30984	1370	(29740-32306)	29197	32599	32010	1644	(31106-33362)	29424	34369	0.2672
	**Average vessels length**	154.80	28.70	(133,6-186,2)	126,7	198,8	163.90	28.24	(143,1-209,8)	130,8	212,2	0.5927
	**Total number of end points**	796.70	84.99	(732,8-845)	708	953	796.50	42.52	(775-821)	712	824	0.9967
	**Lacunarity**	0.09616	0.01934	(0,08132-0,1129)	0,07982	0,1302	0.0851	0.009995	(0,07874-0,0902)	0,07016	0,1013	0.2419
**20 Months**	**Vessels area**	279676	35614	(249563-302187)	219575	322178	283323	12546	(272596-294099)	265677	301433	0.8177
	**Total number of junctions**	512.00	86.99	(461,5-559,8)	406	670	562.30	42.84	(525,8-589,3)	516	638	0.2323
	**Branching index**	0.0005884	0.0001141	(0,000471-0,000683)	0,0004046	0,000685	0.000723	0.00000552 7	(0,0006753-0,0007574)	0,0006652	0,0008212	**0.0264***
	**Total vessels length**	29897	3467	(26649-33092)	24568	33805	30549	1114	(29561-31236)	29038	32293	0.6703

**Average vessels length**	102.80	41.07	(67,83-142,4)	44,75	158,8	145.80	34.43	(114,2-171,4)	95,26	190	0.0779
**Total number of end points**	1056.00	273.40	(803,8-1361)	773	1431	859.30	144.20	(761,3-937,8)	747	1132	0.1510
**Lacunarity**	0.1478	0.06642	(0,09296-0,1981)	0,08899	0,2668	0.09356	0.01204	(0,0854-0,1029)	0,08056	0,1149	0.0773
