# Supplementary material for: Retinal Vascular and Structural Changes in the Murine Alzheimer’s APPNL-F/NL-F Model from 6 to 20 Months
Source: Biomolecules. 2024 Jul 10;14(7):828. doi: 10.3390/biom14070828 (PMC11274728; doi:10.3390/biom14070828)
Supplement: Supplementary file 1 [file biomolecules-14-00828-s001.zip › Supplementary table 4.pdf]

Supplementary Table 4. Analysis date of retinal thickness at the different study times. (WT: wild type, n= 6 for each study group at each time point).

			WT n=6		$APP^{NL-F/NL-F}$ n=6		
		**Sector**	**Mean**	**SD**	**Mean**	**SD**	**P-value**
**6 Months**	**Inner retina**	**N1**	160.20	4.708	150.80	3.601	**0.0065****
		**N2**	157.20	7.333	147.30	3.327	**0.0130***
		**S1**	157.70	5.989	155.30	5.538	0.46103
		**S2**	159.70	4.546	157.70	5.538	0.4567
		**T1**	165.50	5.244	148.80	2.563	**0.0022****
		**T2**	162.30	5.391	149.00	5.621	**0.0043****
		**I1**	163.20	6.911	152.20	2.483	**0.0043****
		**I2**	167.70	9.266	152.50	3.45	**0.0043****
	**Outer retina**	**N1**	83.50	7.765	91.00	4.195	0.0866
		**N2**	89.33	8.501	93.67	2.16	0.2857
		**S1**	88.83	6.145	92.33	5.68	0.4957
		**S2**	93.33	5.574	96.50	3.619	0.3896
		**T1**	79.83	4.491	91.17	2.401	**0.0022****
		**T2**	88.17	6.432	95.67	2.944	**0.0216***
		**I1**	83.83	10.61	89.33	3.777	0.4719
		**I2**	84.33	10.17	88.00	3.098	>0.9999
**9 Months**	**Inner retina**	**N1**	166.00	10.88	155.50	3.834	0.0714
		**N2**	158.20	8.589	154.30	3.882	0.5541
		**S1**	168.50	13.58	160.30	3.559	0.6234
		**S2**	161.80	6.795	162.20	3.189	>0.9999
		**T1**	163.20	7.834	157.00	3.406	0.1428
		**T2**	157.50	4.764	157.80	2.483	0.8550
		**I1**	162.30	2.944	155.50	3.271	**0.0087****
		**I2**	160.00	6.387	157.70	3.559	0.6861
	**Outer retina**	**N1**	84.50	8.55	88.67	4.59	0.2576
		**N2**	89.83	6.432	88.83	5.115	0.7965
		**S1**	88.50	7.688	88.50	7.314	>0.9999
		**S2**	99.17	7.521	94.50	4.764	0.2229

		**T1**	88.17	5.981	87.33	3.445	0.9156
		**T2**	94.33	4.926	89.33	5.241	0.1775
		**I1**	87.83	5.115	86.00	6.782	0.5606
		**I2**	88.67	7.554	83.5	4.278	0.1926
**12 Months**	**Inner retina**	**N1**	162.30	8.454	151.70	4.546	**0.0108***
		**N2**	159.50	7.036	149.80	4.875	**0.0238***
		**S1**	164.50	8.264	157.00	3.521	0.1040
		**S2**	162.50	3.782	156.00	3.464	**0.0216***
		**T1**	161.70	8.71	152.30	3.933	**0.0173***
		**T2**	159.80	5.456	150.50	5.05	**0.0195***
		**I1**	163.20	9.087	153.00	2.828	**0.0108***
		**I2**	165.30	7.763	156.00	4.336	**0.0130***
	**Outer retina**	**N1**	87.67	6.154	92.17	2.401	0.1926
		**N2**	90.50	7.12	95.50	2.811	0.0931
		**S1**	91.00	5.657	94.83	3.43	0.1212
		**S2**	92.83	4.834	100.00	4.817	**0.0476***
		**T1**	89.00	3.847	92.50	2.429	0.1407
		**T2**	90.17	4.262	96.17	3.189	0.0606
		**I1**	89.83	4.491	91.67	2.066	0.1753
		**I2**	86.50	6.473	88.00	3.578	0.5822
**15 Months**	**Inner retina**	**N1**	156.50	4.231	151.00	1.789	**0.0260***
		**N2**	153.50	3.146	150.20	1.835	0.0693
		**S1**	155.80	5.845	156.50	4.416	0.7944
		**S2**	157.30	4.844	157.20	6.178	>0.9999
		**T1**	155.50	5.01	154.00	4.775	0.6147
		**T2**	153.80	3.545	152.50	4.637	0.7403
		**I1**	160.20	3.43	154.00	4.817	**0.0433***
		**I2**	162.20	3.251	157.20	4.401	0.0952
	**Outer retina**	**N1**	91.33	4.719	92.00	3.847	>0.9999
		**N2**	93.17	4.07	93.17	4.708	0.9177
		**S1**	91.67	3.882	94.00	5.177	0.3701
		**S2**	94.00	6.099	95.83	6.242	0.8983

		**T1**	91.33	6.743	94.67	3.933	0.1948
		**T2**	93.17	5.636	97.17	6.94	0.2684
		**I1**	91.00	5.404	94.00	3.406	0.3896
		**I2**	89.50	3.886	91.67	5.574	0.5844
**17 Months**	**Inner retina**	**N1**	154.00	5.177	151.00	1.789	0.2078
		**N2**	152.70	6.713	150.20	1.835	0.0952
		**S1**	159.50	9.247	156.50	4.416	0.6667
		**S2**	160.00	10.39	157.20	6.178	0.7879
		**T1**	155.50	3.937	154.00	4.775	0.5173
		**T2**	155.70	5.574	152.50	4.637	0.2597
		**I1**	154.20	5.776	154.00	4.817	0.6645
		**I2**	158.20	4.875	157.20	4.401	0.7208
	**Outer retina**	**N1**	88.50	3.619	95.50	3.674	**0.0130***
		**N2**	90.50	5.718	97.83	7.083	0.0844
		**S1**	91.67	7.916	96.50	5.612	0.3355
		**S2**	94.67	4.412	98.50	6.535	0.1645
		**T1**	90.17	3.764	96.33	4.32	**0.0368***
		**T2**	93.50	5.089	97.83	5.307	0.2575
		**I1**	89.17	5.231	96.00	3.578	**0.0390***
		**I2**	86.00	3.225	94.17	4.07	**0.0065****
**20 Months**	**Inner retina**	**N1**	156.80	2.787	154.20	6.047	0.1082
		**N2**	150.30	2.503	150.80	2.858	0.8615
		**S1**	152.50	4.97	155.30	2.944	0.1234
		**S2**	154.30	5.086	153.80	2.317	0.6125
		**T1**	160.20	4.875	153.00	3.688	**0.0303***
		**T2**	153.80	3.189	153.5	3.728	0.7835
		**I1**	158.20	5.492	156.00	3.847	0.5844
		**I2**	158.20	3.125	159.00	1.673	0.5086
	**Outer retina**	**N1**	93.17	7.468	91.50	4.183	0.8939
		**N2**	97.67	5.854	95.83	5.115	0.4134
		**S1**	96.33	6.186	93.17	3.971	0.4112
		**S2**	98.33	6.346	95.00	3.688	0.3723

**T1**	89.67	8.981	91.33	6.154	0.4610
**T2**	96.00	6.573	93.67	4.546	0.6125
**I1**	94.67	5.785	92.17	3.061	0.5216
**I2**	92.50	5.958	91.00	2.966	0.8593
