# Supplementary material for: Retinal Vascular and Structural Changes in the Murine Alzheimer’s APPNL-F/NL-F Model from 6 to 20 Months
Source: Biomolecules. 2024 Jul 10;14(7):828. doi: 10.3390/biom14070828 (PMC11274728; doi:10.3390/biom14070828)
Supplement: Supplementary file 1 [file biomolecules-14-00828-s001.zip › Supplementary Table 5.pdf]

OCTA adquisition parameters

OCT Image	OCT Scan Pattern
Scan Angle: 20°	Number of B-Scans: 512
Size X: 512 pixels (2.3 mm)	Pattern Size: 20° x 20° (2.3 x 2.3 mm)
Size Z: 496 pixels (1.9 mm)	Distance between B-Scans: 4 µm
Scaling X: 4.43 µm/pixel	
Scaling Z: 3.87 µm/pixel	
ART Mode: ON (6 images averaged)	
EDI Mode: OFF	

OCT adquisition parameters

OCT Image	OCT Scan Pattern
Scan Angle: 30°	Number of B-Scans: 61
Size X: 1536 pixels (3.4 mm)	Pattern Size: 30° x 25.0° (3.4 x 2.8 mm)
Size Z: 496 pixels (1.9 mm)	Distance between B-Scans: 47 µm
Scaling X: 2.22 µm/pixel	
Scaling Z: 3.87 µm/pixel	
ART Mode: ON (30 images averaged)	
EDI Mode: OFF	
